# Language Barriers and Healthcare Challenges for Immigrants with Limited English Proficiency After Health Reform in the United States

**DOI:** 10.3390/ijerph23010009

**Published:** 2025-12-19

**Authors:** Tiffany D. Joseph, Virginia Martinez

**Affiliations:** Department of Sociology and Anthropology, Northeastern University, 201 Renaissance Park, 360 Huntington Avenue, Boston, MA 02115, USA

**Keywords:** immigrants, healthcare access, language barriers, health reform, ACA, Massachusetts health reform, exclusion

## Abstract

Amid increased immigration enforcement and the rollback of health reform in the United States, immigrants’ healthcare access is at the center of these policy debates. Though previous research has examined the impact of language barriers on healthcare access, few studies have examined it since health policies have been implemented. This article explores how various language barriers undermined Latino/a immigrants and citizens’ ability to access coverage and care under comprehensive health reform in the United States. Specifically, this paper examines how language barriers affect: (1) coverage enrollment experiences; (2) navigation of the US healthcare system; and (3) patient-provider interactions. Using interviews from 209 immigrants, healthcare providers, and immigrant and healthcare advocates in Boston, MA, this article shows that language barriers reduced healthcare access for limited English proficient (LEP) individuals despite health reform in three ways: (1) negatively affected coverage enrollment; (2) made it more difficult to navigate the system; and (3) hindered patient-provider interactions. Nevertheless, respondents described immigrants and advocates’ efforts to reduce those barriers and improve healthcare experiences. This article elucidates our understanding of persistent healthcare language barriers despite reforms to increase healthcare access. This article concludes with a discussion regarding how the current socio-political climate will undermine healthcare access in the United States.

## 1. Introduction

The healthcare system in the United States is notoriously expensive and difficult to navigate, especially for more marginalized populations [1,2]. Furthermore, the expensive cost of health insurance was a primary reason that many Americans lacked access to coverage in the early 21st century [1,3]. Despite those high costs in coverage and care, health outcomes of Americans lagged behind those of their Global North counterparts [1,4]. The implementation of the 2010 Affordable Care Act (ACA) health reform aimed to increase access to health insurance and reduce the country’s high uninsured population [5,6]. Modeled after the 2006 Massachusetts Health Reform (MA), the ACA made health coverage more accessible for low- and middle-income Americans and provided more patient protections that prevented Americans from being denied coverage for pre-existing health conditions. Though increasing access to coverage for an estimated 31 million Americans, the reform explicitly excluded many noncitizens [7,8]. Attempts to repeal and undermine the ACA under the first Trump and second Trump administrations have resulted in more Americans being uninsured [9]. Yet, even among those with health coverage, navigating the US healthcare system remains a challenge. This is especially the case for individuals with limited English proficiency [10,11]. While previous studies have explored how language hinders healthcare access for marginalized populations in the US, few have examined it after US health reform and among more than one language group.

This paper explores how various language barriers undermined Latino/a immigrants and citizens’ ability to access coverage and care under the MA and ACA reforms. Specifically, this paper aims to examine how language barriers affect: (1) coverage enrollment experiences; (2) navigation of the US healthcare system; and (3) patient-provider interactions. Thus, this article elucidates our understanding of how healthcare language barriers persist despite policy reforms to increase healthcare access.

### 1.1. Language Barriers in the US Healthcare System

Language barriers in the US healthcare system persist even though there are legal frameworks in place to guarantee language access in all institutions that receive federal funding [12]. Such frameworks were established to ensure that English speakers and people who speak languages other than English are entitled to equal treatment [12,13]. Specifically, Title VI of the 1964 Civil Rights Act explicitly prohibits discrimination based on race, color, or national origin in federally funded institutions and programs. In that law and under President Bill Clinton’s Executive Order 13,166 in the year 2000, language was tied to one’s national origin. Thus, noncompliance with Title VI and Executive Order 13,166 violates the civil rights of LEP individuals in institutions lacking sufficient language access. More recently, the 2010 ACA included a lesser-known provision that prohibited language-related discrimination in healthcare settings. However, most of these legal frameworks have not been fully enforced, which means that language barriers continue to undermine LEP individuals’ healthcare experiences [10,11].

Previous research indicates that language barriers in the healthcare system exist in multiple ways. These barriers affect patients’ understanding of the healthcare system, making it difficult to navigate the system. LEP individuals experience increased difficulty making appointments, booking follow-up appointments, refilling their prescriptions, and adhering to their treatment plans [11,14,15,16]. As a result, LEP individuals are less likely to have health insurance coverage, use preventative services, and see healthcare providers consistently [14,17,18]. Language barriers also influence LEP individuals’ communication and relationships with healthcare professionals, ranging from physicians to office staff [10,11,14,16]. A consequence is that LEP patients experience frustration and report increased dissatisfaction, mistrust in healthcare providers and the system, and perceived discrimination in healthcare settings [8,14,17,18]. Cumulatively, these barriers contribute to LEP patients experiencing delayed care and lower quality care relative to their English-speaking counterparts, which negatively influences their health outcomes [12,13,19,20]. Such outcomes are made worse from the inability to receive timely and preventive care, which can lead to emergent medical conditions [18,19].

In the absence of institutional language access, LEP individuals sometimes mitigate language barriers through the use of ad hoc interpreters, like friends, relatives, and bilingual individuals who can translate for them [11,19,21]. While these ad hoc interpreters provide vital support and increase their relatives’ sense of comfort, such interpreters contribute to more challenges and healthcare risks [15]. Because these ad hoc interpreters are not trained in medical terminology or confidentiality concerns, they are unable to effectively serve as language mediators between patients and providers [11,19,20,21]. Thus, language barriers undermine healthcare access and contribute to negative health outcomes for LEP individuals.

### 1.2. Health Reform Efforts in Massachusetts and the US

Massachusetts became the first US state to implement comprehensive health reform in 2006, extending health coverage eligibility to all state residents regardless of their documentation status. The reform successfully reduced the state’s uninsured population and became the model for the 2010 federal Affordable Care Act [22]. However, due to longstanding federal legal status restrictions, the ACA excluded most immigrants from key provisions [23]. Such restrictions undermined health coverage gains for Massachusetts immigrants under the state’s original reform.

State legislators revamped the reform to minimize the number of immigrants who could lose coverage after the ACA implementation in late 2013. But documentation status still stratified their coverage, with undocumented immigrants having the fewest options [8]. They also were prohibited from purchasing private health coverage in the state’s health insurance exchange. Massachusetts maintained its Health Safety Net (HSN) program, which provided primary care to undocumented and lower-income residents, but it could not be used at all healthcare facilities. Though documented immigrants had more options, those with certain types of visas and legal permanent residents for fewer than five years could not benefit from the Medicaid expansion, one of the ACA’s key provisions [8,24]. Consequently, Massachusetts created a state-funded coverage option for those immigrants. People with legal permanent residency for at least five years and naturalized citizens were eligible for both public and private coverage options.

The ACA’s most well-known provisions extended access to health coverage by expanding Medicaid eligibility for lower-income Americans and created health insurance marketplaces where middle-income Americans could purchase private coverage with government subsidies [25,26]. Young adults up to age 26 could remain on their parents’ health insurance plans and no one could be denied coverage due to pre-existing health conditions [25,27]. A lesser-known provision prohibited language-related healthcare discrimination. Specifically, healthcare organizations had to provide adequate access for people with limited English proficiency through creating language access plans, ensuring sufficient medical interpreters for those who needed them, and posting notices about the availability of qualified interpreters [14]. As of 2023, such changes were yet to be fully implemented. Between President Trump’s 2025 executive order making English the official language of the United States and passage of the One Big Beautiful Bill Act, which will significantly reduce access to health coverage, it is unlikely this ACA provision will not be enforced [28,29].

## 2. Materials and Methods

Data in this article come from a larger qualitative study conducted by the first author that examined how shifts in state and national-level health policy reconfigured healthcare access for Latino immigrants in Boston, Massachusetts. Boston was an ideal site because of its ethnoracial diversity, sizeable immigrant population, and location as capital of the first U.S. state to implement comprehensive health reform that became the model for the 2010 ACA. The first author interviewed 209 people comprising Brazilian, Dominican, and Salvadoran immigrants, healthcare providers, and employees from immigrant and health advocacy organizations from 2012–2019 (Table 1).

Interviews were conducted at three different time periods coinciding with health policy shifts: (1) 2012–2013 after the Massachusetts health reform implementation, (2) 2015–2016 after ACA implementation, and (3) 2019 under the first Trump Administration, that aimed to repeal the ACA and implemented rigid immigration policies, creating a chilling effect in immigrants’ healthcare access. For example, the US legislature passed the 2017 Tax Cut and Jobs Act which ended the ACA’s tax penalty for the individual mandate requiring health coverage. This research design allowed for an examination of intersections between city, state, and federal policies, alongside health, immigration, and welfare policies which perpetuated exclusion in the healthcare system.

Immigrants described their barriers obtaining health coverage and navigating the healthcare system. Brazilians, Dominicans, and Salvadorans represent the largest immigrant groups in Boston (the first author recruited Brazilian and Dominican immigrants in 2012 but added Salvadoran immigrants in 2015 with the influx of Central American immigrants to the US-Mexico border, some of whom settled in Boston). Brazilians are typically undocumented, from middle-class backgrounds and racialized as White. Dominicans are usually legal permanent residents, from lower-class backgrounds and racialized as Black. Salvadorans are eligible for temporary protected status which grants eligibility for some public benefits, from lower-class backgrounds, and racialized as Mestizo or Brown. These three groups are racialized as “Latino” in the United States despite their heterogeneity in nationality, language, culture, physical features, and legal status. Their unique backgrounds provide relevant insights into how Latinos’ differences influence their health care. See Table 2 for demographic information about the immigrant sample.

Table 3 shows the self-reported English proficiency of immigrant respondents. Most of the immigrants reported their proficiency as good or average. Of the three groups, Brazilians were more likely to describe their proficiency as excellent, while Dominicans and Salvadorans reported lower levels of proficiency.

Healthcare professionals from the Boston Health Coalition (BHC) with immigrant patients described how shifts in MA and ACA policies influenced their ability to care for their patients. BHC is a pseudonym for the hospital network, which offers quality care to underserved populations, has three hospitals, and operates 15 ambulatory care offices providing primary and specialty physical and mental health services. Lastly, immigrant and health advocates highlighted greater Boston’s sociopolitical climate for immigrants, explaining different factors and laws that shaped immigrants’ healthcare access, and connected immigrants with health and social services. For example, undocumented immigrants could not obtain driver’s licenses during this study. This made accessing care difficult despite the state’s inclusive health policy.

To recruit respondents, the first author posted multilingual fliers in Brazilian, Dominican, and Salvadoran organizations and at BHC healthcare facilities. The first author also volunteered at immigrant advocacy organizations to learn about these communities’ challenges, which aided in recruitment for the different stakeholder groups. The first author also used purposive snowball sampling, commonly used for difficult-to-recruit groups [30,31]. The first author recruited and interviewed new participants across the different stakeholder groups each year of this study. The first author continued conducting interviews across each group until there was sufficient overlap in participants’ responses regarding immigrants’ healthcare experiences. This aligns with what qualitative scholars refer to as “saturation” in line with the grounded theory approach [32,33,34]. The one exception was in 2019 for the immigrant sample, which was much smaller, as recruitment was more difficult due to the socio-political climate under the first Trump administration. Finally, to ascertain the impact of policy shifts throughout this study, the first author reinterviewed 11 providers and 10 advocates. But amid concerns about immigrant respondents’ safety, all of them were new each year of this study, none were reinterviewed.

For the immigrant sample, the first author recruited immigrants of any documentation status who had been in the US for at least one year and ranged from ages 27–64. People in this age group were not eligible for Medicare (individuals 65 years and older) or ACA coverage provisions for children and young adults up to age 26. The first author recruited a range of BHC providers (e.g., physicians, medical interpreters, staff) who primarily had immigrant patients. Finally, the first author recruited immigrant and health advocates who worked for organizations that directly provided assistance and support for immigrant communities to access the social safety net (e.g., health coverage, food assistance). This recruitment strategy was effective despite the intensifying racialized and anti-immigrant climate during the project. Because this qualitative study did not involve a large random sample, the findings are not generalizable to broader populations. But this study design contributes important insights into challenges other marginalized communities might encounter navigating the US healthcare system. This study was granted human subjects research approval for the duration of this study from the first author’s university.

The first author conducted interviews with immigrants in Brazilian Portuguese or Spanish that lasted for 60 min. These interviews explored immigrants’: (1) demographic background in the US and home country; (2) journey to the US; (3) access to and experiences with health coverage and healthcare system in the US and home country; (4) perspectives and experiences regarding discrimination in the US and home country; (5) racial and skin tone classifications in the US and home country; (6) quality of life in the US; and (7) knowledge of health policy and socio-political shifts in the US.

Interviews with BHC providers were typically 60 min, conducted in English, and explored: (1) types of health services provided; (2) demographics and health coverage of patients; (3) impact of racial, language, and cultural differences on patient-provider interactions; (4) common physical and mental health issues and challenges among immigrant patients; (5) perspectives regarding discrimination among immigrants; and (6) role of policy and socio-political climate changes in their ability to treat immigrants.

Lastly, interviews with immigrant and health advocates were generally 60 min, conducted in English, Spanish, or Portuguese, and examined: (1) organization’s mission, types of services offered, and populations served; (2) challenges immigrants face accessing health coverage and care; (3) impact of racial, language, and cultural differences on interactions with immigrants; and (4) role of policy and socio-political climate changes in affecting organization services for immigrants.

All except for two of the 209 interviews were audio recorded with consent from the respondents. Two immigrants declined recording amid fear about what might happen to the recording. After conducting each interview, the first author wrote fieldnotes summarizing major themes, interesting comments, and body language or gestures. A professional transcription company transcribed the recorded 207 interviews. The first author reviewed each transcript while listening to the audio recording to ensure accuracy and consistency between the audio recording and transcript. Then, the revised transcripts were formatted for data analysis in NVivo qualitative software version 15.

For analysis of findings presented in this paper, the authors used an inductive, grounded theory approach to develop themes from a systematic analysis of the data [32,33,34]. We used open and focused coding, reading each transcript closely and developing an extensive list of recurring themes, with one- to three-word phrases describing various aspects of immigrants’ healthcare access as related to policy, health coverage, and experiences with healthcare providers in the US and their home countries before and since migrating to the US [34]. It was from this coding process that the authors developed language barrier-related codes: (1) level of English proficiency; (2) language and health coverage enrollment experiences (e.g., completing paperwork); and (3) language and navigating healthcare system experiences (e.g., determining where and how to use coverage, make appointments); and (4) language and patient-provider interactions (e.g., miscommunication, use of interpreters). Next, we placed related interview transcript portions under these codes and created sub-codes that corresponded to each stakeholder group to compare perceptions between the immigrant, healthcare provider, and advocate stakeholder groups. We also included separate subcodes for Brazilian, Dominican, and Salvadoran respondents to examine commonalities and differences among the three groups. This was important because Brazilians speak Portuguese, Dominicans speak Spanish, and Salvadorans speak Spanish and Indigenous languages. We analyzed each interview in the language in which it was conducted to minimize losing nuances in translation. Only the quotes appearing in this article were translated from the original transcripts. We continued this exhaustive process until all interview transcripts were analyzed, which led to the results presented in this article.

## 3. Results

Analysis of the qualitative data revealed that language barriers negatively shaped healthcare access in three ways. First, they deterred coverage enrollment due to lack of appropriate assistance and resources for patients with limited English proficiency. Next, language barriers made it difficult for patients to navigate the healthcare system. Finally, these barriers hindered patient-provider interactions and sometimes led to longer waiting times for patients. While advocates and BHC providers were more likely to discuss language barriers related to enrollment challenges, immigrant respondents more frequently described how language barriers affected their navigation of the healthcare system or patient-provider interactions. We explain each of these findings below in detail.

### 3.1. Enrollment Materials

Despite living in Massachusetts where all income-eligible residents were theoretically able to apply for health coverage, enrollment processes were not easy. The process entailed filling out detailed paperwork online and submitting necessary proof of state residence and income to determine one’s possible coverage options. This already difficult process was cumbersome for English proficient residents, but even more so for those with limited English proficiency. Interviews with respondents across multiple stakeholder groups reiterated this complexity and how language barriers sometimes resulted in people remaining eligible for coverage but being unable to enroll in coverage.

Quinn, an employee from the state’s health insurance office who was interviewed in 2019 explained that MA residents still struggled applying for coverage. The state had worked diligently to streamline the enrollment process since the original implementation of the 2006 reform. But Quinn acknowledged that the different eligibility rules under both the 2006 state and 2010 ACA reforms and insufficient language assistance made it hard for applicants to understand the extensive paperwork from the state years after both reforms were implemented:

I think it’s challenging for people to understand… how to interpret all the various rules that are associated with that [enrollment]. And then beyond that, how to understand the reams of documentation [mail] they will see [receive] from the state, which we provide, because we think it’s helpful and is important for legal rights, of course. But also, it can be overwhelming. It can be in English and some are in Spanish as well. And in addition to that we have what we call Babel sheets, that are provided in different top languages on how to get language assistance. But you know, as you could imagine if that’s [in] the end of the packet, it can be fairly difficult [to find].

Multi-lingual assistance from the state government to enroll in coverage was very limited throughout this study. So, health advocacy organizations like the one Edward worked for simplified the process through a dedicated helpline that people could call to obtain enrollment help in various languages. In a 2013 interview, Edward described how his organization met this huge need for an increasingly diverse Massachusetts population:

We have a helpline to get hundreds of calls a day in English, Spanish and Portuguese and in fact more than half of our calls are not in English… [we] help people navigate the system and figure out to how enroll, what are the rules, what are the complicated rules, including about immigration status and how that affects your application. We fill out applications over the phone we mail [it] to them [patients], they sign it and they mail it back to us. We make sure to be perfect to be send it in [to the state] and then we call them [patients]… we also help people who are just lost in the system, don’t understand how to use their health care and so on.

The mission of Edward’s organization is to reduce language barriers to enrollment so that those who call can successfully apply for coverage and use it to access care. While this is a very helpful service, MA residents must learn about it through their existing social networks, usually from family members or connections to immigrant advocacy organizations. I heard from Edwards’s colleagues, at the same organization and advocates from other organizations, that people reach out to Edward’s organization for enrollment assistance after trying unsuccessfully to enroll in coverage until a relative or friend recommends this organization.

Once people successfully applied for coverage on their own or with assistance from Edward’s organization, they received mail notifications from the state in English or Spanish, which notably created language barriers for individuals who spoke other primary languages. When this study began in 2012, those were the only two language options available. Gloria, a BHC provider interviewed in 2013 explained how resulting language barriers led to her patients bringing those state coverage notifications to her for deciphering:

The majority of people receive the [coverage] notification in English. And if they have written in the application that they speak Spanish, [the state] is doing a good job sending notification in Spanish. But I haven’t seen any other languages… However they insert a page with a lot of different language[s] and say if you need—to translate this notification–but it’s pretty generic and sometimes even providers and the patients, [it] is confusing to understand what they[notifications] are saying… It’s difficult. I help them to read. It’s because of English [as] a second language… And [even] native English speaker[s] come to ask my help to read the letter because, they are not easy [to understand].

Gloria’s quote alludes to the challenges of language barriers in two ways that were commonly mentioned throughout this study. First, those with primary languages other than English could not understand enrollment materials and coverage notifications. But the second language barrier also speaks to the healthcare-related jargon that makes comprehension difficult even for those whose primary language *is* English. Though we discuss how these language barriers shape navigating the system and engaging with providers later in this paper, it also negatively affects coverage enrollment, which is the entry point to the US healthcare system.

The confusing mail notifications that Gloria described were sent after initial enrollment but also were mailed before patients re-enrolled in their coverage, usually on a yearly basis. If patients did not return necessary paperwork or moved without notifying the state to receive reenrollment notifications, patients could lose their coverage. Beyond this general reenrollment process, patients had to deal with changes to the healthcare system as the 2010 Affordable Care Act was implemented, starting in late 2013. This created more confusion, made more cumbersome for those with language barriers. An employee named Maggie from another health advocacy organization described how she saw this play out among patients she worked with in 2015 after ACA implementation in Massachusetts:

I think particularly, immigrant populations, I noticed that when reading through call notes, a lot of our callers will still check regularly to see if they have to reapply. And so they don’t know really the rules on if they need to reapply. And with changes to the system... I mean, we saw before, the Massachusetts Health reform and people needing to enroll in a [healthcare] plan, that was particularly a problem for people who spoke English as a second language. And we still see that with the new program [under the ACA reform in MA]. So, people who don’t know that they’re eligible for this program don’t know to enroll in a plan… the notices still, they only go out in English or Spanish, and only they’re listed if they want notifications in Spanish. So that continues to be an issue. And the website, the online application isn’t available in [multiple] languages.

Therefore, language barriers complicated (re)enrollment procedures for health coverage throughout this study, but especially amid policy shifts from the original MA reform to the ACA implementation. Lack of sufficient language assistance from state healthcare bureaucrats meant that LEP individuals were at more of a structural disadvantage when applying for coverage, which they were theoretically entitled to as residents of the state. Such structural disadvantages in language access bureaucratically disentitled these individuals from coverage.

### 3.2. Language as Deterrent to Navigating the US Healthcare System

For individuals who successfully enrolled in coverage, language barriers subsequently posed challenges to navigating the US healthcare system to obtain care. Respondents from the various stakeholder groups described how those barriers posed challenges to finding providers who accepted their coverage, medical appointments over the phone, or refilling prescriptions. Kevin, a BHC doctor interviewed in 2012 described how one of his Brazilian patients made medical appointments under the MA reform:

Just like last week someone was telling me about how they couldn’t, a patient of mine, I’ve been calling him to see me and follow-up. He said that he calls, and he couldn’t even do anything. Basically, I have a bunch of patients who feel like because of the wait time or lack of people even picking up the phone and then language problems once they do, that they have to physically show up at the office to get any business done. Like if they want a visit appointment, get a refill or you name it. Now that’s a subsegment of people with no English proficiency and no one who can help them who is English proficient. [But] I think the majority of people have enough English proficiency to manage asking the front desk staff if they can have [an] interpreter to talk to the nurse. But there are a few people that can’t even get that far and can’t figure out, can’t even schedule appointments over the phone… We might have like two of eight who speak Portuguese… So when they[patients] come [to the office], they know who speaks Portuguese, they go up to them and talk to them about it.

Kevin’s quote adeptly describes how language barriers affect navigation of the US healthcare system. Even though the Title VI of the 1964 Civil Rights Act is supposed to ensure language access for those who need it at federally funded institutions like healthcare facilities, this was not the reality in greater Boston’s healthcare settings. BHC has a reputation for providing quality care to all patients and an institutionalized medical interpretation system, consisting of on-call medical interpreters.

The first author interviewed some of those interpreters for this study, who described taking pride in their ability to dismantle some of the language barriers that BHC patients experienced navigating the healthcare system. At the same time, they described challenges in doing so, such as difficulty mediating patient-provider conversations, burnout from being in high demand for interpretation services, the impact of different interpretation modalities (in person versus phone versus video), and navigating language dialect differences during interpretation. For example, Adriana, a Portuguese BHC interpreter interviewed in 2013 explained how her workload increased after the Massachusetts health reform increased demand for healthcare services. Before the reform, she did more in-person face-to-face interpretation in the room with providers and patients. But increased demand and cost of in-person interpretation led BHC to shift to telephone and video interpretation, which contributed to Adriana’s job fatigue:

I am afraid, it’s very risky, the life and health of patients. My workload seeing 40 patients a day concerns me. I cannot continue like this. We [interpreters] have a 30-min lunch break and you can take 15-min breaks. Our workload is very heavy… Before the reform and all this technology [phone/video interpretation], when I did face-to-face [in-person interpretation], I could walk back to my office and have a little time [to decompress between interpretation sessions]. But now that we are here [in office with video/phone interpretation], you end one call and go right to another one. I think they [BHC administrators] are reducing face-to-face [interpretation] a lot.

Adriana recognized the health implications of her fatigue for patients and how insufficient time for breaks or to decompress between patients was taking its toll. While medical interpreters provided a vital service to make health care more inclusive, patients also noticed the demand that Adriana alluded to. Some immigrant respondents shared that needing an interpreter often increased their wait times to see a provider. Rosalicia, a Brazilian immigrant interviewed in 2013, described this experience before her English proficiency improved:

Yes, language, if don’t know how to express yourself [affects your healthcare access]. When you don’t, when I didn’t speak English, it took longer [to get appointments] because, there wasn’t as much access to interpreters. Today, I don’t need it [interpreters] because I can communicate much better.

While increased wait times for interpreters and insufficient language access made it difficult to navigate the healthcare system under the original MA reform, these language barriers did not improve after the ACA implementation. If anything, language access demand increased for preventive and specialty care as more people gained access to coverage. But language barriers undermined their ability to effectively use that coverage. Madison, a BHC provider interviewed in 2016, shared her observation that LEP patients are unable to receive adequate care from an on-call physician if health issues emerge after hours:

They certainly don’t know how to–often to continue following up with the specialist. I’ve also noticed so, we have a doctor that’s on call every evening… and I seldom, almost never get a page from a phone call from a patient who doesn’t speak English at night. And even on Saturdays I’ve hardly ever see[n] patients who don’t speak English. And I think maybe they don’t know, or I think they don’t feel comfortable calling after hours. I honestly don’t know like what happens if you’re a Spanish-speaking patient and you call our office after hours and the answering service picks up. Like, do they offer you an interpreter? I feel like I’ve never had to call back a patient on pager who didn’t speak English.

Madison’s quote indicates her recognition of this healthcare gap and how it leaves LEP patients at a disadvantage. Inadequate language access potentially puts their health at risk. Josefina, a Dominican health advocate interviewed in 2019 succinctly described language barriers as a challenge for Dominicans navigating the system in the last year of this study:

They [Dominicans] encounter so many problems but the biggest one is language, the language barrier when they go to hospitals where there is no [medical] translation and also access to medicine.

Therefore, although health reforms increased access to health coverage, they did not similarly increase the resources needed to ensure adequate language access for LEP individuals to use their coverage to obtain health services.

### 3.3. Patient-Provider Interactions

Another way that language barriers shaped healthcare access was through patient-provider interactions, positive or negative. Respondents across all stakeholder groups explained how limited English proficiency reduced patient comfort with and trust of providers. This could occur in basic medical appointments or patients had emergent health issues. Forty-six of the 82 immigrants interviewed who reported their English proficiency as average or not proficient and reported needing an interpreter in healthcare settings. Of these, the majority reported positive experiences despite having a language barrier. This was particularly the case if an interpreter was available and the provider worked well with that interpreter. For example, Elena, a Dominican immigrant interviewed in explained:

Very good [my healthcare experience with my doctor]. She is very conscious, a very good person. They [doctor and interpreter] are very professional. When I do not understand things [with my health], they try to find a way to help me understand.

From the provider perspective, even when there were positive experiences with LEP patients, providers and interpreters could not always be there to help patients. One BHC provider, Aubrey who was interviewed in 2019, described how language barriers continued to affect her interactions with patients, especially when they were not in her presence. Aubrey’s ability to communicate with patients in multiple languages put patients at ease, but:

Yes, I, I speak Portuguese and French and some Arabic which is very, very helpful. People feel more comfortable talking to me… but I think also, it’s a barrier still there. I never know, what’s gonna happen after they leave [the] room with me (laughs). You know, like, you set up a test, you give them phone calls, but they might miss the test, they miss the studies... And I don’t think we do a great job of communicating with them… For example, in the clinic where I work, most providers, if they don’t speak the language of the patient, they will pull up the lab results, they’ll send a message to call a patient interpreter… But the letters [lab results] are in English.

Aubrey’s quote demonstrates yet again how language barriers complicate patients’ navigation of the US healthcare system. While there may be interpreters available for actual appointments, such interpreters may not be with patients when they receive lab results over the phone or in their online medical charts. Aubrey’s quote also shows that while some patient-provider interactions may be positive despite language barriers, at some point, the inability to effectively communicate can negatively shape those interactions, their ability to navigate the healthcare system, and ultimately patients’ health.

Various immigrant respondents described negative patient-provider interactions they personally experienced or observed. Silvana, a Brazilian immigrant interviewed in 2015, was fluent in English but went with other Brazilians to healthcare settings in her capacity as a social worker for a local organization. She explained:

I have accompanied patients with language [barriers] to their appointments. And the doctor doesn’t have patience. We see that there are many healthcare professionals without cultural sensitivity. So, they can be more negligent, with less patience. The problem is greater for immigrants that do not speak English, they feel intimidated and vulnerable. This results in a cascade effect where people don’t get the care they deserve.

For Silvana, it is not just language barriers that affect such interactions. But less patience and cultural sensitivity from providers can result in substandard care. Patients’ lack of comfort in such settings over a prolonged period can contribute to negative outcomes. The first author heard something similar from Camila, a BHC medical interpreter quoted earlier. She spoke about her in-person interpretation with a diabetic LEP patient in another part of our interview:

When you are face-to-face [interpretation] and you can see the expressions on both faces [doctor and patient], then you can tell, ‘oh there is an issue here, wait a minute, what questions [do] you have for him [the doctor].’ We have had patients that have had diabetes up to the 800 number [glucose level]. He [ a patient] was dizzy, his vision was blurry, he wasn’t seeing right. [The patient said] “And I am sorry, how much did you say?” [I said] “It’s 900.” [The patient said] “Is that low or is that high?” And it gets to point that you are really like, “well sir, do you know anything about diabetes?” [The patient said] “No.”

In this case, the inability of Camila’s patient to obtain sufficient medical interpretation in previous healthcare visits affected his navigation of the system and interactions with providers to the extent that his diabetes spiraled out of control. This led to delayed care that resulted in his unusually high glucose level, which can result in a diabetic coma. And it was clear in this encounter that the patient did not fully understand that he had diabetes or how to manage it because of previous challenges navigating the system to obtain necessary care.

Finally, an immigrant advocate, Lidia who was interviewed in 2019, described a client who had birth complications that worsened because of language barriers and providers not effectively listening to the patient:

She’s telling me the story of when she gave birth and the complications that she had and her trying and going back and forth between doctors before finally, she switched to a different hospital. And she was like, “I’m not getting [good] service here. I’ve been saying something’s not right. Something’s not right… Like my doctor, my gynecologist is saying, “you’re fine, you’re fine, you’re fine.” … She was feeling like she was going to die. And so yeah, when she went to switched to a different hospital, they like ran some tests and they’re just like, “okay, something is wrong with your uterus.” And so, she had gone untreated for months after she had her child…and her English at that point, just like was very limited, and she’s like, “I’m trying to do this with the newborn in my hands, and I had no idea what to do. And no one was listening to what I was saying.” … And she’s like, she knows that that’s a form of discrimination.

Thus, negative patient-provider interactions stemming from language barriers had severe health consequences for patients.

### 3.4. Agency in Reducing Language Barriers

Respondents in the different stakeholder groups described various ways that language barriers shape LEP patients’ healthcare access in the previous section. But they also outlined efforts to reduce those barriers for immigrants to obtain care. Some immigrant respondents shared that they actively sought out healthcare facilities with sufficient medical interpreters or providers (co-ethnic or not) who spoke their primary language. Patient-provider language concordance added a level of comfort and confidence in their interactions and ability to navigate the system. Marcia, a Brazilian advocate shared in 2013:

I think that many [Brazilians] experience discrimination, I have been discriminated against in hospital many times. That’s why people always go where [hospitals] there are interpreters, where Brazilians work, where there are Brazilian doctors, they feel more comfortable.

Similarly, a Dominican immigrant named Victoria, interviewed in 2012, explicitly sought out Hispanic providers for the same reason:

I always try to go to Hispanic clinics where there are Hispanic doctors who understand my culture and that I can relate better to. They can understand me, my way of thinking. I think the best part is, they provide better service.

While immigrants used their agency to reduce language barriers in health care, some health advocates’ organizations provided services with a similar goal. Joshua, a healthcare advocate does trainings to improve healthcare literacy in immigrant communities. In his 2015 interview, he described the content covered in those multi-lingual trainings with community partners:

What we talk about is where should you seek care, and so there is a big thing where people end up going to the emergency room when they have a headache or stomachache, things like that, really encouraging people to seek care at Community Health Centers, clinics, places where they can get cheaper care and perhaps better care and have to wait less time for relatively minor health care situations. We talk about what is health insurance, what is a health plan, what does that mean, what is a co-payment. This is a very important issue for people to understand… I am actually collaborating with ESOL [English Speakers of Other Languages] programs and teachers in order to develop this training in a way that people can access it better and understand it better.

The work of organizations like Joshua’s and medical interpretation services at BHC and other healthcare facilities demonstrate their recognition of how language barriers must be overcome for LEP immigrants to effectively navigate the system and receive medical care. Immigrants too, used their agency to find trainings like Joshua’s or providers and healthcare facilities where their primary language was spoken. Immigrants exercised such agency to receive better healthcare treatment and feel more empowered in healthcare contexts.

## 4. Discussion

Language barriers created significant challenges for immigrants to receive health care in greater Boston. Those barriers occurred at all stages of engaging with the healthcare system, first in the coverage enrollment process, next in navigating the system to use their coverage, and finally in their interactions with providers. Despite living in Boston, the birthplace of comprehensive health reform with its world-class healthcare facilities, respondents from the different stakeholder groups described how having limited English proficiency complicated individuals’ healthcare experiences. Some of these challenges remained even in healthcare settings with a deep infrastructure for medical interpretation like BHC, where a range of providers and some medical interpreters were interviewed throughout this study.

The findings in this paper overlap with those of previous studies on language barriers among LEP patients more generally, but also specifically among Brazilians and Dominicans, who are understudied in healthcare research. Lindsay et al.’s study of Brazilian immigrants reported that the inability to communicate with providers in Portuguese was a significant healthcare barrier and, in some cases, resulted in hostile encounters with healthcare staff in Massachusetts [18]. Escobedo et al.’s study of Dominican immigrants in New York revealed that language barriers cultivated dissatisfaction and mistrust of the healthcare system [10]. This study contributes to previous research by demonstrating that comprehensive health reform at the state and federal levels did not eliminate healthcare language barriers, undermining access to care for LEP individuals.

State and federal health policy changes that occurred over the duration of this study were meant to increase access to health coverage so that more individuals could gain access to more affordable and preventive care. In Massachusetts, the percentage of residents insured has remained steady at about 98% since the 2006 reform [35]. The remaining uninsured tend to be immigrants and LEP individuals, which was the demographic of interest in this study [35]. In general, the complex options and eligibility criteria to apply for coverage created immense administrative burdens in coverage enrollment under the original Massachusetts Reform and after the Affordable Care Act in Massachusetts and for people across the country after ACA implementation [8,24,36]. Thus, despite having the highest percentage of insured individuals nationally, Massachusetts patients continue to report challenges using their coverage to obtain care and navigating the system, challenges made more difficult for LEP individuals [8].

The language barriers described in this paper indicate how the MA and ACA health reforms could not fully address pre-existing problems in the healthcare system. While both increased access to coverage, language barriers hindered increasing access to care for LEP individuals. The ACA’s extra funding to increase language access in federally funded healthcare facilities did not sufficiently improve such access. Furthermore, ACA compliance measures that aimed to assess language access in healthcare settings were not effectively enforced. For the LEP respondents in this study, their civil rights regarding language access as outlined in Title VI of the 1964 Civil Rights Act have been violated. This means that their language barriers will persist.

Nevertheless, despite the challenges language barriers posed for respondents in this article, some used their agency to reduce such barriers for themselves or others. Immigrants unable to access medical interpreters in some healthcare settings sought out healthcare facilities with interpreters or providers who spoke their primary language. Medical interpreters in such facilities went above and beyond to help patients navigate such settings and receive the care they needed. And organizations worked to fill the language access gaps in the system through setting up helplines to assist patients with enrollment, teach them health literacy, and explain how the US healthcare system worked. These strategies empowered immigrants and eased patient-provider interactions so that patients could receive quality care. Given that MA and ACA health policies could not fully provide language access, immigrants, providers, and organizations stepped in to do what policy effectively could not. Moving forward, these efforts will remain vital to create inclusive healthcare access in Boston and elsewhere in the United States.

This study has many limitations, notably that this study was conducted among a small sample of immigrants, providers, and advocates in a major US city. This means the findings are not generalizable beyond the sample. Other immigrant groups in Boston or across the country may have more nuanced experiences with language barriers in the healthcare system. Nevertheless, the findings presented shed important insights regarding how language barriers may hinder healthcare access for the broader population in other parts of the United States after the passage of comprehensive health reform. Other studies cited previously in this paper demonstrated similar barriers prior to and since the implementation of the ACA [10,11,12,13,16,18,19,20,21,22]. The fact that such language barriers endure is a testament to how the healthcare system has yet to meet the needs of a linguistically diverse US population.

Language barriers in healthcare access for limited language proficient immigrants also reveal consistent findings and gaps globally. Though the research presented focuses on language barriers in the US healthcare system, similar results have been found in other Western countries. Immigrants’ limited language proficiency also poses a structural barrier to healthcare access in Canada and Western Europe [11,37,38,39,40,41,42]. In Canada, language barriers increase difficulty finding providers and contribute to ineffective patient-provider communication and delayed access to time-sensitive care [37,38,39]. In Spain, France, and the United Kingdom, immigrants’ language barriers also contribute to perceived discrimination based on their ethnicity, in turn, resulting in unmet healthcare needs, poor quality care, limited availability of intercultural mediators [11,40,41,42]. It should be noted that this collective research highlights immigrant groups from many countries. But it does not specifically address Latin American migrants’ language barriers, which are the key focus of this paper. 

The socio-political climate in the United States is now shifting in ways that will likely worsen language barriers and healthcare access for LEP individuals and the general US population. President Trump has signed executive orders making English the official language of the country, ending diversity, equity, and inclusion (DEI) initiatives, and increasing draconian immigration enforcement measures. Perhaps most consequential, he has signed into law funding cuts to federal healthcare programs like Medicaid. Cumulatively, these legislative actions undermine language access and make it more difficult to provide inclusive health care. Furthermore, immigrants are more fearful of obtaining health care and millions of people are projected to lose their health coverage. Simply put, the US healthcare system is changing significantly. The negative health impacts will not only affect those with limited English proficiency, but anyone who will need medical care at some point.

## 5. Conclusions

This article explores how language barriers undermined immigrants’ healthcare experiences after the passage of health reform (Massachusetts and the Affordable Care Act) from 2012 to 2019. Drawing from interviews with 209 immigrants, healthcare providers, and immigrant and healthcare advocates in Boston, MA, this article showed that language barriers undermined healthcare access for limited English proficient (LEP) individuals through inadequate language access in: (1) coverage enrollment processes; (2) navigating the healthcare system; and (3) patient-provider interactions. Nevertheless, respondents described immigrants and local organizations’ efforts to reduce those barriers.

## Figures and Tables

**Table 1:** Immigrants, Providers, and Advocates Interviewed in 2012–2013, 2015–2016, and 2019 (Total *N* = 209).

Stakeholder Group	Pre-ACA 2012–2013	Post-ACA 2015–2016 ^a^	Post-2016 Election: 2019 ^b^
**Immigrants**	N = 31	N = 39	N = 12
Brazilians	21	15	8
Dominicans	10	14	2
Salvadorans	N/A	10	2
**Healthcare Providers at Boston** **Health Coalition**	N = 19	N = 19	N = 14
Physicians	5	6	5
Medical Interpreters	4	4	2
Other Medical Staff	10	9	7
**Immigrant/Health Organizations**	N = 20	N = 25	N = 30
Brazilian	6	4	7
Dominican	2	4	2
Salvadoran	N/A	2	2
General Immigrant Organizations	3	5	9
Health Organizations	9	7	8
City/State Officials	0	3	2
**Total**	70	83	56

a. Salvadorans added to the 2015–2016 sample amid political debates about Temporary Protected Status renewal. b. 2019 Immigrant Sample is smaller due to recruitment difficulty in socio-political climate.

**Table 2:** Demographic Characteristics of Immigrants Interviewed in 2012–2013, 2015–2016, and 2019 (N = 82).

Demographics	2012–2013 Immigrant Sample (N = 31)		2015–2016 Immigrant Sample (N = 39)			2019 Immigrant Sample (N = 12)	
	Brazilians (N = 21)	Dominicans (N = 10)	Brazilians (N = 15)	Dominicans (N = 14)	Salvadorans (N = 10)	Brazilians (N = 8)	Dominicans (N = 2)
Gender (number of women)	12	5	8	10	6	5	2
Median Age (years)	40	55	43	56	40	46	55
Average Time in US (years)	12	14	10	21	19	11.5	28
Average Individual Monthly Income	$3969	$480	$1720	$843	$1383	$3017	$3050
Employed at Time of Interview	16	6	11	8	8	6	2
Customary Occupations	Hairstylists, Childcare, Cleaning, Education	Childcare, Cleaning	Childcare, Cleaning, Landscaping	Childcare, Restaurants	Childcare, Clerical, Restaurants	Cleaning, Nurse Assistant	Dentist, Housecleaning
Documentation Status (at interview)							
Undocumented	6	3	6		6	5	
DACA						1	
Tourist Visa (B1)	2		1				
Religious Visa (R1)	1						
Student Visa (F1)			3				
Work Visa (H1A/B)	1		1				
Temporary Protected Status (TPS)	N/A	N/A	N/A	N/A	1		
Green Card/LPR	10	4	3	11	2		
Naturalized Citizen	1	3	1	3	1	2	2
Health Coverage Type							
Uninsured (N)	1		2	2	3	1	
Public Coverage (N)	12	8	10	10	7	3	2
Private (N)	8	2	3	2		4	2

**Table 3:** Immigrants’ Self-Reported English Proficiency 2012–2013, 2015–2016, and 2019 (Total N = 82).

Nationality	Pre-ACA 2012–2013	Post-ACA 2015–2016	Post-2016 Election: 2019
**Brazilians**	N = 21	N = 15	N = 8
Excellent	5	3	1
Good	11	3	1
Average	3	5	3
Not Proficient	2	4	3
**Dominicans**	N = 10	N = 14	N = 2
Excellent	0	2	2
Good	4	1	0
Average	4	6	0
Not Proficient	2	5	0
**Salvadorans**		N = 10	N = 2
Excellent		0	1
Good		2	0
Average	N/A	7	0
Not Proficient		1	1
**Total**	31	39	12

## Data Availability

Given the vulnerability of the study population and IRB protocol, data for this article is not publicly available.

## Abbreviations

The following abbreviations are used in this manuscript:

LEP	Limited English Proficiency
MA	Massachusetts
ACA	Affordable Care Act
HSN	Health Safety Net
